# Role of Chitosan Hydrogels in Clinical Dentistry

**DOI:** 10.3390/gels9090698

**Published:** 2023-08-29

**Authors:** Suraj Arora, Gotam Das, Mohammed Alqarni, Vishakha Grover, Suheel Manzoor Baba, Priyanka Saluja, Saeed Awod Bin Hassan, Anshad M. Abdulla, Shashit Shetty Bavabeedu, Shahabe Saquib Abullais, Gurparkash Singh Chahal, Anchal Ohri

**Affiliations:** 1 Department of Restorative Dental Sciences, College of Dentistry, King Khalid University, Abha 61321, Saudi Arabia; maalqarny@kku.edu.sa (M.A.); baba@kku.edu.sa (S.M.B.); samhasan@kku.edu.sa (S.A.B.H.); sbavabeedu@kku.edu.sa (S.S.B.); 2 Department of Prosthodontics, College of Dentistry, King Khalid University, Abha 61421, Saudi Arabia; 3 Department of Periodontology and Oral Implantology, Dr. H. S. J. Institute of Dental Sciences, Panjab University, Chandigarh 160014, India; vishakha_grover@rediffmail.com (V.G.); gpchahal29@gmail.com (G.S.C.); ohrianchal1993@gmail.com (A.O.); 4 Department of Dentistry, University of Alberta, Edmonton, AB T6G 2P5, Canada; priyanka.salujaarora@gmail.com; 5 Department of Pediatric Dentistry & Orthodontics, College of Dentistry, King Khalid University, Abha 61321, Saudi Arabia; anshad@kku.edu.sa; 6 Department of Periodontics, College of Dentistry, King Khalid University, Abha 61421, Saudi Arabia; sshahabe@kku.edu.sa; 7 Department of Periodontics, Datta Meghe Institute of Higher Education and Research, Deemed to be University, Wardha 442001, India

**Keywords:** biopolymer, chitosan, cross-linking, hydrogel, regeneration, tissue engineering

## Abstract

Biopolymers are organic polymers that can be treated into intricate designs with porous characteristics that mimic essential biologic components. Due to their superior biosafety, biodegradability, biocompatibility, etc., they have been utilized immensely in biomedical engineering, regeneration, and drug delivery. To obtain the greatest number of results, a literature search was undertaken in scientific search engines utilizing keywords. Chitosan is used in a variety of medical sectors, with the goal of emphasizing its applications and benefits in the clinical dental industry. Chitosan can be dissolved in liquid form and combined with other substances to create a variety of products, including fibers, hydrogels, membranes, microspheres, resins, sponges, pastes, tablets, and micro granules. Chitosan has been studied in a variety of dental applications. Chitosan is used in the prevention of caries and wear, in pulpotomy to accelerate osteogenesis in guided tissue regeneration due to its hemostatic property, and primarily to benefit from its antimicrobial activity by adding it to materials, such as glass ionomer cement, calcium hydroxide, and adhesive systems. With its antibacterial activity and biocompatibility, chitosan is leading the pack as a promising ingredient in the production of dental materials. The current review provides an update on the background, fundamentals, and wide range of uses of chitosan and its gels in dental science.

## 1. Introduction

Chitin is one of the naturally found polymers like collagen, alginate, and cellulose [1]. The recent surge in the research on these polymers is based on their ability to be used as an alternative to fossil fuels and being environmentally friendly [2,3,4]. Chitin occurs in the skeleton of arthropods, the cell wall of fungi, insects, and mushrooms and appears primarily as waste from the seafood/fish industry [2,3,4,5,6,7,8,9,10]. The limited de-acetylation of chitin converts it into chitosan, which otherwise has a limited existence in the environment. The term chitosan is used for deacetylated chitin, which contains 60% D-glucosamine residues minimally [11,12]. The deacetylation converts chitin, the water-insoluble polymer, to chitosan, which is partially water-soluble [13]. It is preferred to investigate the characteristics of the final product as the polymer may undergo many changes during the process, and it is difficult to estimate its final structure and the properties it shall achieve after the completion of the manufacturing. Chitosan can be mixed with different components in liquid form and molded into various shapes: fibers, hydrogels, membranes, microspheres, resins, sponges, pastes, tablets and micro granules [9,12].

Chitosan has been utilized in dentistry for caries prevention, as well as in nano-materials to increase mechanical integrity, antimicrobial previously damaged tissue regeneration, dentin matrix, and to close the canal space during root canal therapy. Chitosan nanoparticles are resorbable films that can be used to administer antibiotics (such as metronidazole, chlorhexidine, and nystatin) to periodontal tissues in situ, therefore preventing fungal infections and oral mucositis [14,15]. Chitosan has been identified as a promising substrate material for periodontal tissue regeneration due to its compliance with the aforementioned features. Thambiliyagodage et al. reported on a commercially available non-fluoride chitosan-based dentifrice and found a considerable decrease in tissue loss. A number of chitosan-based restorative formulations have been investigated and are being considered for the effective delivery of organic amelogenin at the location of enamel defects in order to achieve human enamel regeneration [16]. A number of researchers have found that covering dental implants with chitosan has encouraging outcomes. Recent progress in this subject has resulted in the use of chitosan as a carrier for chitosan-mediated stem cell repair [14,15,16].

Knowledge of the structure of hydrogels and the mechanism of gelation of intelligent hydrogels is essential to designing bioinspired hydrogels [17,18]. As one of the raw materials in hydrogels, chitosan has been highly pursued due to the polymer’s biocompatibility, biodegradability and low toxicity. Its good biocompatibility is well documented in animal studies, which implies its usage for the fabrication of implantable biomaterials [18,19]. The current review aims to provide an update on the structure, properties and numerous applications of chitosan as a frontline biomaterial in various dental procedures.

## 2. Historical Perspective of Chitosan

Chitin was discovered by Braconnot in 1811 from fungi and was named as fongine. Braconnot elaborated that it contained a high proportion of nitrogen, primarily acetate of ammonia contaminated with oil. Acetic acid was formed from this fraction after treatment with concentrated sulfuric acid. Odier, in 1823, isolated an analogous compound from the elytra of insects after they were treated with hydroxide solutions at high temperatures. He coined the name ”chitine/chitin”, rooted in the Greek word chitos, referring to tunic or envelope. Rouget discovered the compound in 1859 by utilizing a hot potassium hydroxide solution. Later, Gilson identified the occurrence of glucosamine in it, and concurrently, a similar term, “Chitosan”, was used by Hopper-Seyler [20,21,22,23]. Structural analysis of chitin and chitosan was conducted in the 1930s by diverse techniques, including X-ray diffraction, enzyme-related methods and infrared spectroscopy. In 1936, chitosan was first used in the making industry, and two patents regarding the production of chitosan from chitin, films and fiber fabrication from chitosan were obtained by Rigby [22].

## 3. Structure of Hydrogel Chitin

Hydrogel chitin and cellulose are polymeric compounds of monosaccharides made up of-(14)-2-acetamido-2-deoxy—D-glucopyranose and -(14)-2-deoxy—D-glucopyranose units, respectively, they are structurally similar. The N-acetyl glucosamine units that makeup chitin are typically depicted as long-chain homo-polymer or poly (N-acetyl-D-glucosamine). The three polymorphic forms of chitin discovered through X-ray diffraction research are α, β, and γ chitin. The varied properties and functions of chitin are determined by its many molecular configurations. α-chitin, which has its N-acetyl glucosamine chains organized in antiparallel directions, is the steadiest and strongest form of chitin. In contrast to β-chitin, which has its chains arranged in a parallel orientation, γ-chitin has two of its chains with the same polarity and one of its chains with the opposite polarity [22,23,24]. Chitin is insoluble in most of the organic solvents due to its rigid crystalline structure. However, it can be dissolved in a calcium chloride dehydrate methanol (Ca solvent) solvent system. The α- and β-chitin hydrogels can easily be developed using the Ca solvent system. Using these hydrogels, it is able to develop scaffolds and membranes for a variety of biomedical applications, such as tissue engineering and wound dressing [25]. Hydrogels can be similar to the extracellular environment of human tissue; hence, they are capable of being used in biomedical applications. In addition to being biocompatible and nontoxic, it has an excellent ability to exist in a multitude of physical forms, e.g., nanoparticles, nanofibrils, microspheres, composite gels, fibers, films, etc. These unique biochemical properties are of immense use for a wide variety of applications for human health [22,24].

## 4. Production of Hydrogel Chitin

Many different life forms, including insects, fungi, mushrooms, and some aquatic creatures, have chitin as a constituent of their biomolecule. However, for commercial production, the majority of raw biopolymer is extracted from sea crustaceans because marine biowaste is a major resource for the mass synthesis of chitin and chitosan. It is available in enormous quantities and at a low cost as a byproduct of the seafood processing industry [9,12]. The mineralized shells contain 15–40% chitin along with two other biological compounds, i.e., Calcium carbonate (20–50%) and proteins (20–40%). Because different species affect the quality and freshness of the shell and the season when it is harvested, the amount of chitin greatly varies. Additional sources of chitin include clams and oysters [13,14,15,16,17,18]. The mushrooms serve as a better resource as these are cultivable and offer a more controlled production and a safer product than the animal source, e.g., seafood [14]. The chitosan produced from the mushrooms exhibits a small molecular mass disparity and degree of deacetylation in comparison to the product obtained from seafood. Deproteinization, demineralization, and decolorization are the three key stages that make up the extraction of chitin [20]. Acids are used to remove inorganic components in the conventional chemical process, strong inorganic alkalis are used to extract proteins (often at 50–60 °C), and oxidizers are used to remove color. These procedures often entail the use of sodium hydroxide to break down proteins in order to extract lipids and pigments (melanins, carotenoids) and hydrochloric acid to lyse the salts, especially calcium carbonate and calcium phosphate, leaving behind a colorless substance. It is crucial to take into account the acid content, contact time, and temperature to reduce hydrolytic and thermal degradation, along with associated chemical changes [3,11,21,22,24].

**Figure 1:**
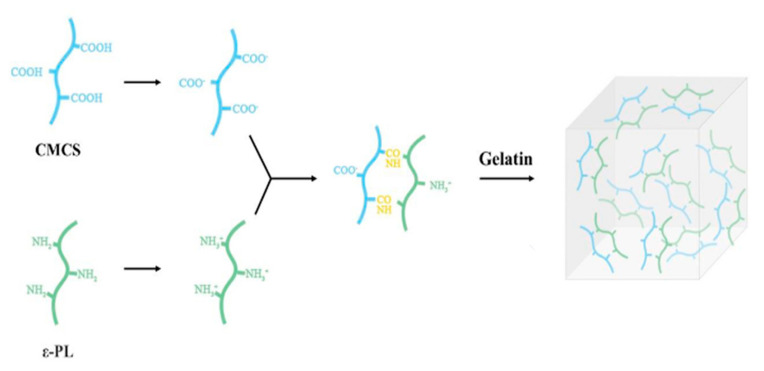
An injectable hydrogel used in dentistry applications consisting of polylysine (ε-PL) andcarboxymethyl chitosan (CMCS) adapted from Ref. [26].

Both homogeneous and heterogeneous deacetylation processes can be used to create chitosan hydrogel, as shown in Figure 1. The heterogeneous approach, which involves amorphous parts of the polymer reacting without affecting the crystalline region, is frequently employed in businesses. In order to deacetylate chitin and create chitosan, the acetamide groups are typically hydrolyzed using concentrated NaOH or KOH (40–50%) at temperatures exceeding 100 °C. [18,19].

### Properties of Chitosan

Chitosan presents a multitude of characteristics in terms of physical–chemical, biological, and technological aspects. In fact, chitin is the sole naturally occurring biopolymer. The compound exhibits a polycationic property at low pH (below 6.3); however, with pH rising above 6.3, chitosan’s amine groups lose protons and acquire reactivity. With acetylation well below 50%, it is soluble in all water-base media having low pH. There is a great range of solvents for the compound, including dilute inorganic acids, concentrated H_2_SO_4_, organic acids, and additional organic compounds, e.g., tetrahydrofuran, ethyl-acetate, 1,2-dichlorethane etc. [27]. The most popular acids for chitosan solubilization are acetic and formic. Because it depends on a number of factors, including acetylation, ionic concentration, solution pH, and the protonation acid used, solubility is a fascinating but difficult property to control [19,23]. Additionally, the location of acetyl groups throughout the macromolecular structure influences the ability of chitosan to dissolve, depending on the circumstances surrounding its manufacture. Due to the fact that chitosan can dissolve and then precipitate into a wide range of physical shapes, including beads, films, membranes, fibers, or nanofibers, the protonation reaction is particularly significant for chitosan. In addition, it can be cross-linked to create materials like fibers or sponges that can be used in a variety of ways [28,29]. Chemical treatments to enhance cross-linking with epoxides or glutaraldehyde result in more stable configurations of the molecule. Chitosan is comparatively more workable than its parent compound, chitin, for such chemical modifications, but the resulting compounds from chitosan typically have relatively lesser stability due to its greater hydrophilicity and sensitivity to pH [30].

Chitosan is a nontoxic and biodegradable polymer that can attach to microbial and mammalian cells. It helps in bone formation as it increases osteoblast formation along with a rejuvenating reaction on the gum’s connective tissue [30]. It demonstrates good biocompatibility with endothelial, epithelial, myocardial, chondrocytes, hepatocytes, fibroblasts, and keratinocytes [19,30,31]. It exhibits antitumor, anticholesteremic, hemostatic, fungistatic, immunoadjuvant and antibacterial features [30,31,32]. The positively charged reactive functional group of amino in chitosan helps prevent plaque formation [33]. Though insoluble at alkaline and neutral pH, it forms water-soluble salts with organic and inorganic acids. It becomes positively charged upon dissolution in acidic media. The change in the pH, degree of deacetylation and ionic strength can change its properties (solubility, pKa) [34].

Every deacetylated unit in chitosan has an amino group at the C-2 position, and every repeat unit has primary and secondary hydroxyl groups at the C-6 and C-3 positions, respectively. To enhance compatibility and its properties, these reactive groups can be easily chemically derivatized under benign circumstances. Quaternized chitosan, for example, has the potential to be a mucoadhesive and permeability-enhancing absorption enhancer across the intestinal epithelium [35,36]. Cyclodextrin-linked chitosan sounds promising from the perspective of pharmaceutics, which covers drug delivery, aesthetics, and analytical chemistry [33]. The hydrogen bonds between the molecules of chitosan are damaged by the presence of positively charged ions, which causes them to dissolve in water. Chitosan’s solubility is primarily dependent on the molecular weight and the degree of deacetylation; with partial removal of the acetyl groups, solubility in water increases, and biodegradability and biocompatibility increase. The antibacterial and anti-biofilm actions of chitosan, as well as its solubility and viscosity, are significantly influenced by the above-mentioned factors. [34,35,36]. One idea holds that it destroys cells by pushing Ca++ out of the anionic sites in the membrane [37]. Some oral microbes, such as *Prevotella intermedia*, *Porphyronomonasgingivalis*, and *Actinobacillusactinomycetemcomitans*, show strong antiplaque activity in response to it. The bacterial cell wall may be destroyed by the positively charged amino groups, NH_4_, interacting with the negatively charged, electrostatically charged surface of the bacterial cells. The cellular contents may leak owing to alterations in the cell membrane permeability. Chitosan has been found to have an antibacterial effect on oral microorganisms. Numerous studies support the use of chitosan as an antibacterial ingredient in dental materials, composites, and oral hygiene products [37,38].

Chitosan is subjected to chemical modifications in order to enhance its solubility, rheological characteristics, thermal stability, and oxidation resistance. The active groups in chitosan’s chemical structure are amino groups and hydroxyl groups at the C_3_ and C_6_ locations. Because of the free rotation, the NH_2_-amino group is typically more reactive than the C_6_-OH main hydroxyl group, with the secondary C_3_-OH hydroxyl group being less reactive than the primary hydroxyl group [36,37,38,39,40]. Chitosan can be chemically modified on the amino, hydroxyl, or both amino and hydroxyl groups to create derivatives that are N-, O-, or N, O-modified. On hydroxyl groups, etherification, esterification, crosslinking, graft copolymerization, and O-acetylation are performed, whereas on amino groups, acetylation, quaternization, Schif’s base reaction, and grafting are performed [34,35]. Chitosan-based hydrogels are generally temperature reversible and pH-sensitive, which swell in acidic pH and shrink in basic media. The abundant availability and its remarkable properties, including antimicrobial activity, mucoadhesive properties, biodegradability, and biocompatibility, make it an appealing molecule for a variety of applications, such as drug delivery, photodynamic therapy, and blood anticoagulation. Additionally, the positive charge of the compound helps to stimulate cellular biological activity, interaction and differentiation, applications in the field of tissue engineering, wound healing, and wound dressings [41,42].

Numerous free amino and hydroxyl groups found in the chitosan backbone could be exploited as active sites, and various methods for building chitosan-based nanomaterials have been documented. NPs, nanogels, micelles, liposomes, nanofibers, and nanospheres are a few examples. The drug delivery method has been thoroughly researched for oral and injectable administration, topical delivery, colon-targeted drug delivery, cancer therapy, vaccination and gene transfer by utilizing the nanomaterials produced from this compound [39,40,41,42,43]. The drug release from chitosan-based dosage forms depends on the physicochemical characteristics of the encapsulated drug, such as whether it is hydrophilic or hydrophobic, partitioning efficiency, size, dose, etc., polymer characteristics, such as bioadhesion to mucin or skin, swelling and gel-forming ability in various body fluids with various pH and ion concentrations, as well as the presence of co-polymers and excipients. The following mechanisms are involved in the drug release from chitosan-based dosage forms: diffusion, swelling, erosion, biodegradation etc. [41,42,43,44].

## 5. Applications in Dentistry

Owing to its properties like antimicrobial, bioactivity, and biocompatibility, it is considered a prospective biomaterial for dental applications [38,39,40], which are summarized in Table 1.

### 5.1. Preventive Dentistry

#### 5.1.1. Dentifrices

For preventive measures, it could be used as a component of mouthwash, a component of toothpaste against dental plaque, and a component of toothpaste against erosion abrasion and mucoadhesive-cariostatic substance delivery systems [40]. The dentifrices are the mainstay in daily plaque control and good oral hygiene on a daily basis. They have a pivotal position in preventing the demineralization of the tooth by various caries promoting foods and drinks. There have been many studies that documented the combination of chitosan with various other ingredients normally used in dentifrices, such as NaF, SnCl_2,_ KNO_3_, HA, and SnF_2,_ with increased effectiveness of the dentifrice in some of the studies [41,42]. In their report on the commercially available, fluoride-free chitosan-based dentifrice, tissue loss was significantly reduced, according to Ganss et al. Using NaF- and Sn-based dentifrices has also been linked to similar results in terms of halting the deterioration of the dentin matrix and enamel [43,44,45]. These results are explained by the strong affinity of chitosan for binding to zeta potential-negative structures like enamel and salivary pellicles, as well as the cationic character of chitosan in combination with a low pH. Thus, over mineralized surfaces, a shielding multilayer organic matrix would form [5]. By adjusting environmental stimuli, including temperature, pH, magnetic field, and light, it is possible to regulate the drug loading and release rate of hydrogels. Hydrogels that respond to stimuli can be intelligent drug delivery systems for the controlled, sustained, and targeted release of drugs. Several strategies, including controlled swelling, chemically controlled release, and environmental release, are used to regulate the hydrogel drug release behavior. These days, smart hydrogels are also being developed as drug delivery systems, including pH, temperature, and magnet-responsive hydrogels [12,19,26,43] The dual-pronged anti-erosive and anti-abrasive properties of chitosan improved the efficacy of Sn2+-based dentifrices to stop tissue loss in acidic oral environments. [41]. Fluoride-containing chitosan nanoparticles were studied by Ebrahimi et al. for their effectiveness in living things. Through tripolyphosphate nanoparticle ionic gelation, sodium fluoride was added to chitosan. Zeta potential, particle size, loading capacity, encapsulation effectiveness, and Fourier Transforms Infrared Spectroscopy were used to characterize nanoparticles. Fluoride and chitosan were cross-linked with tripolyphosphate to create chitosan/fluoride nanoparticles. According to some, the fluoride/chitosan nanoparticles created in the study may be a potential way to distribute fluoride for the prophylactic use for dental caries [46]. Plaque builds up as a result of oral germs adhering to tooth surfaces. It is thought that electrostatic and hydrophobic interactions are what cause the germs and tooth surfaces to stick together. Because the positively charged amine group in chitosan derivatives competes with it, these interactions are prevented. The anionic sites and carboxylic acid residues present in bacterial cell walls may interact electrostatically with the amine groups in chitosan to provide antibacterial activity [33].

The most recently used gelling agents, such as sodium alginate mucilage, Irish moss, and tragacanth, were only used in toothpaste to the extent that they could gel and due to their carbohydrate composition, they also required antimicrobial preservatives. Chitosan, on the other hand, possesses antibacterial qualities and works well as a gelling agent without the need for preservatives [33,38].

#### 5.1.2. Remineralization Potential

Hydrogel chitosan is a bioactive substance in and of itself that aids in the remineralization of dental hard tissues. Its functional groups may encourage apatite nucleation, which may precipitate the mineralized tissue. As a biomimetic system, it functions. The long-term continuous release of bioactive compounds is made possible by the polycationic mucoadhesive polymer known as chitosan. It degrades biologically and produces non-toxic metabolites. A large amine group at the C-2 position also allows structural changes in the individual monomeric units of the molecule. The compound has evolved hugely as a remineralization agent for the dental tissues viz enamel and dentin. The therapy of white spot lesions with chitosan may be performed after the application of bioactive glasses alone or when they are used in combination with a polyacrylic acid slurry. It was discovered that the subsoil contains more minerals and that chitosan increased the ability for remineralization. [43]. Resende et al. formulated toothpaste with biosurfactants, and either sodium fluoride or fungal chitosan was tested for cytotoxicity, antibacterial activity, and inhibitory potential against *Streptococcus mutans* biofilm. When compared to the tested commercial toothpaste, their compositions had equivalent effects on S. mutans’ cellular viability in the biofilm. The current findings demonstrated that, in comparison to commercial toothpaste, the suggested formulations are promising [47,48]. Several restorative formulations utilizing chitosan have been investigated and are currently being evaluated in order to successfully transport organic amelogenin to the region of enamel defects. Recently, amelogenin was dispersed using a chitosan hydrogel to reestablish the aligned crystal structure. Chitosan has the dual advantage of avoiding secondary caries and not interfering with the alignment of the enamel crystals, thanks to its antibacterial properties. Further well-controlled and long-term clinical research in the fields of tissue engineering, biomolecules, and materials science is necessary for better understanding and application of chitosan [42].

Phosphorylated chitosan nanocomplexes and ACP (amorphous calcium phosphate), commonly known as Pchi-ACP, were used to remineralize the demineralized enamel. Another amelogenin-chitosan (CS-AMEL) hydrogel biomimetic system demonstrated that the direction of crystal growth and mineral imbibition were significantly affected by the viscosity of chitosan, which was increased from 1 to 2%. This resulted in the production of huge irregular mineralized accreations resembling enamel crystals [42]. On the other hand, mineralization of dentin is a differential process as compared to enamel; many investigations have been carried out in this regard as well. Carboxymethyl chitosan (CMC) in another research study was revealed to have similar effects and strengthened the structure of dentin [43].

Anti-Inflammatory: Since inflammation is the primary immune response of the body against microbial infections, most oral diseases, including periodontal disease, are usually countered by the inflammatory response of the host. Chitosan and its derivatives have a significant bearing on the process of inflammation. N-acetyl glucosamine is known to stimulate the inflammatory cells, for example, neutrophils, macrophages and even the tissue-resident fibroblasts, in a lot of laboratory investigations. Chitosan particles have inhibited the growth of periodontal pathogens, viz. *porphyromonasgingivalis* and *aggregatibacteractinomycetemcomitans*. The compound further exerts its anti-inflammatory activity by impacting the prostaglandin E2 levels through the JNK pathway. Another investigation revealed many bioinflammatory compounds, such as fibronectin, interleukin-6 and IL-1 beta genes, were also the targets to be influenced by chitosan particles [44,45,49]. Recent work has evaluated the use of a chitosan brush in the nonsurgical treatment of residual periodontal pockets and revealed an equal or better patient outcome in terms of clinical results [45,48,50].

### 5.2. Restorative Dentistry

#### 5.2.1. Hemostasis and Pulpotomy

Bleeding is one of the most common consequences and complications encountered in surgical treatment methods, and effective management of uncontrolled bleeding is a key challenge in healthcare. Hydrogel chitosan tends to interact with blood plasma and erythrocytes to promote hemostasis in fresh, sharply debrided wounds. A large number of chitosan-based hemostatic application technologies have come into being and are continually researched for technical advancements [5,44,46]. Many delivery formulations, like gels, fiber, dressings etc., are commercially available for such applications. One such product, Celox (SAM Medical Products, Newport, OR, USA), is a simple and reliable method for bleeding control. The underlying mechanism is the reaction of the positively charged chitosan particles with the negatively charged human blood cells when the polymer comes in direct contact with blood. Another product in the form of granules has also been utilized to form a cross-linked clot very independent of the natural cofactors, in fact, in combination.

It has also been utilized with sterile saline in the case of pulpotomy of deciduous teeth to obtain hemostasis and enhance the development of the reparative dentin and hard tissue. Thus, it seems to be a good potential material to be used in pulpotomy [6,51,52].

#### 5.2.2. Improvised GIC with Chitosan

Chitosan is a useful addition to traditional glass ionomer cement (GIC), as it causes a significant rise in the release of a variety of cell signaling peptides and growth factors, particularly useful for vital pulp therapy [7]. In an earlier investigation, the impact of chitosan nanoparticles added to GIC (NCH-GIC) content was compared to that of traditional GIC (TGIC) on its mechanical properties and fluoride release. Chitosan fillers in the nanometer range were used. Because chitosan was added, NCH-GIC had much greater bending resistance than TGIC. There was also a concomitant increase in fluoride release throughout seven days in the case of NCH-GIC than in TGIC. Thus, the authors of the investigation reported that the addition of nano chitosan improves the tested properties of the GIC in terms of its anticarcinogenic potential, mechanical properties, and high-resistance applications [34]. Another study exploring the safety and toxicity profile revealed that chitosan-modified GIC demonstrated it to be nontoxic on pulp cells when compared with TGIC. Thus, chitosan-modified GIC may be opted as a material of choice for bioactive dental restorations for various varieties of pulpal and regenerative endodontic conditions [7].

Calcium hydroxide is a widely used medicament and material in restorative dentistry, whose properties could be enhanced with the addition of Chitosan nanoparticles (CNP). When this material was explored to eliminate bacterial biofilms, it could prove much better in the elimination of microbes, both in long and short-term exposures and aided in improving the antibacterial properties of calcium hydroxide [35].

#### 5.2.3. Adhesion and Dentin Bonding

There is published evidence in the context of chitosan used in the “etch and rinse” adhesive system, which adds to the longevity of dental restorations. Hydrolysis of exposed collagen can lead to degradation of the dentin-resin contact and a decrease in bond strength. When dentin is treated with chitosan and an etch-and-rinse or self-etch adhesive system is used, the bond strength is improved while the longevity of the contact is preserved [46]. The use of chitosan antioxidant gel on dentin decreases permeability and related dentin hypersensitivity, as well as has the potential to strengthen the binding between composite resins and the dentin structure. In reality, antioxidant hydrogen has been compared to the standard phosphoric acid etching process, and it has been found that this method of application greatly enhances the link between the restoration and tooth structure [46,50,53].

Researchers are interested in the dentine-restoration interface and bond strength endurance. The dentine replacement materials now available suffer from drawbacks such as acid etching procedure discomfort and smear layer removal difficulty. When the smear layer is not completely removed, the resin monomer frequently does not penetrate well, creating an unstable hybrid layer that is vulnerable to nano leakage. Antioxidant chitosan hydrogels containing propolis, β-carotene, and nystatin were studied and found to provide solid dentine bonding systems with a corresponding improvement in shear bond strength [42].

Generally, all recorded parameters of mechanical properties were observed in higher values for chitosan-based adhesive systems when compared to the conventional contemporary systems used in adhesive dentistry [7]. A research study demonstrated the reduction in the destruction of collagen and deterred the water permeability of the surface layers, thus making the restoration dentin interface more resistant to the ingress of microbial contamination [41]. However, there are contrasting observations also, as in another experimental setup, chitosan-based adhesion did not affect the microbial contamination, particularly against *S. mutans* and *L. casei* and performed poorer as a barrier with respect to the traditional 2-stage adhesive system. Further, there is some previous evidence documenting the impact of chitosan on the bonding strength of the restorative materials with dentin. There was no difference observed in the shear bonding strength of the restorative surface based on the concentration variation of chitosan in the material between 0.12% and 0.25%. However, it was observed with the addition of a higher concentration, i.e., 0.5% and 1% chitosan, there was a significant decrease in the bonding [45,47].

#### 5.2.4. Regenerative Dentistry

Bone repair, Guided tissue regeneration and tissue scaffold functional reconstruction of lost and damaged body parts, especially bony tissue, have led to the search for novel biomaterials for bone healing. Many biological and chemically produced materials have been extensively researched for guided regeneration of bone and also to act as scaffolds for tissue engineering [43]. Usually, these scaffolds have to have chemical and biological properties and characteristics conducive for cellular and tissue growth to be utilized successfully in this area. Chitosan, having a very safe, noncytotoxic, biocompatible, biodegradable and bioactive profile, appears to be an appealing alternative for regenerative medicine. It aids in the attachment, growth and differentiation of bone cells that are osteoblasts and also helps in the laying down of mineralized bone matrix. Further, it can be complemented with a variety of other materials, such as ceramics and polymers already being used in bone regeneration and yield better mechanical and biological properties in combinations. The polymer can be morphed in diverse forms such as sponge bead membranes and nowadays is also being utilized in the nano polymeric forms for optimizing its properties and better workability. Chitosan itself has antimicrobial properties, which tend to further enhance the benefits and predictability of bone regeneration during early wound healing periods of bone defects implanted with bone regenerative materials [4,46,53]. Chitosan’s biodegradability and biocompatibility allow it to be used as a biomaterial in hard tissue repair procedures. It operates on the idea of creating temporary scaffolding in artificial bone while waiting for the implant to resorb and be replaced by natural bone [54]. The mechanical properties of chitosan, which include H-bonds, chains, and crosslinkings, as well as NH2+ with negative tissues in the body, are significantly influenced by its chemical arrangements. This results in good stability and a framework for the formation of new osseous cell growth in cases of wound healing, repair and regeneration. Numerous studies have been conducted, and the results show that chitosan’s characteristics have a significant influence on bone repair and regeneration. According to certain research, chitosan in the form of a sponge activates bone cells and potentiates bone formation [41]. As a scaffold, it helps to keep the clot stable, serves as a surface for bone cell seeding and permeation, and finally resorbs at the location to make room for newly forming bony tissue. When creating a scaffold, certain treatments can be used to modify the chemical properties of the polymer, such as hydrogen bonds and crosslinks in the tissue lattice, to better suit the needs of a particular bone healing scenario [40]. According to a paper by Klokkevold, there has been a larger increase in osteosynthesis and documented increased osteoblastic activity. Chitosan is pliable and helps osteoblastic cells to grow [47].

## 6. Wound Healing

It is crucial to the immune system’s effective operation before and after surgery. Immunomodulators control the body’s ability to fight against certain illnesses. Chitosan contains immunomodulatory properties that cause macrophages to produce proinflammatory cytokines, which in turn hasten the formation of fibroblasts and have an impact on the structure of collagen [54,55]. Chitosan increases the manufacture of hyaluronic acid and extracellular components related to scar formation by releasing acetylglucosaminidase N as a byproduct of hydrolytic and enzymatic breakdown. Following chitosan application, wounds showed increased levels of collagen and osteopontin, as well as a significant infiltration of inflammatory cells. The degree of chitin and chitosan deacetylation (DD) determines the cicatrizing capacity. Because chitosan has a greater DD than chitin, it appears to be a more potent activator for fibroblasts and poses greater resistance to bursting wounds [41,56].

Chitin, a significant component of the outer shells of crustaceans, is the source of the natural biopolymer chitosan. This substance is well-known in the field of wound treatment for its hemostatic qualities. Additionally, it has additional biological functions and influences macrophage activity, which promotes faster wound healing. Additionally, it has the ability to promote tissue organization and cell proliferation. For the treatment of wounds, biological qualities, such as bacteriostatic and fungistatic capabilities, are especially helpful. There are numerous references on chitosan’s use in treating wounds, just like alginate material. In comparison to traditional gauze treatment, novel chitosan-alginate polyelectrolyte complex (PEC) membranes in animal model studies described much better performance of the material in terms of healing of the incisions. In comparison to wounds treated with Opsite1, PEC membrane-treated wounds had a similar closure rate and look. In comparison to the untreated controls, the photo-cross-linkable chitosan hydrogel application over full-thickness skin wounds created on the backs of mice dramatically caused wound contraction and expedited wound closure and healing [48,57,58]. Chitosan was used to study healing at split skin graft donor sites by covering half of the area with the substance and the other half with a regular dressing. It showed how chitosan accelerated wound re-epithelialization and nerve regeneration in a vascular dermis. At the sites where chitosan was applied, quicker recoveries to normal skin tone were seen. Chitin and chitosan therapy showed a significant reduction in treatment time with minimal scar development in several animals [2,59,60,61].

It has been discovered that the metabolites of chitosan breakdown are neither poisonous nor allergic. Additionally, it has been shown that chitosan monomers can stimulate the healing of tooth pulpal insults and act as a matrix support for the healing of dental pulp cells. Collagen is frequently added to chitosan in order to enhance its biological characteristics [4].

### Implant Dentistry

In recent years, there has been a lot of emphasis on the improvisation of the surface of dental implants to enhance the possibility of osseointegration and their longevity in the oral cavity. Being a very amenable, biodegradable, safe polymer, chitosan has also been explored on these lines. Various investigations have reported that the coating of chitosan leads to changes in the surface interactions with the bone bed and the surface characteristics of the implant surface. The coating tends to affect the elastic module at the surface, thus disseminating the stress onto the bone-implant surface more conductively. These coatings are also utilized as portals for the local application of a variety of antimicrobial agents to control infection around the implant osteotomy. A chitosan coating of dental implants has shown promise in several investigations [51,52]. In 2020, Park et al. created a hybrid dental implant using graphene and chitosan (GC hybrid implant) [57]. Under ideal circumstances, the GC hybrid implant (i.e., 1% GC hybrid implant) might considerably boost bone cell proliferation while lowering bacterial activity and the production of biofilms. They offer a logical concept for producing dental implants made of hybrid graphene, utilizing a simple spin-coating method. The created hybrid dental implant was shaped into graphene-containing structures that offered sufficient surface qualities for osseointegration promotion, such as improved wettability and roughness. We also show that the hybrid implant’s altered surface qualities can regulate the actions of bacterial and mammalian cells, presenting a fresh concept for a dental implant [62,63,64,65,66,67]. Further, some of the recent systematic reviews of chitosan use in dentistry are compiled in Table 2 [54,55,56].

## 7. Conclusions

The findings of this study indicate that chitosan is a safe substance to use; nonetheless, a number of difficulties required additional investigation. Various alterations to the chitosan structure have been made in the future to broaden the spectrum of uses and add new aspects to the development of chitosan-based biomaterials. Chitosan is frequently used in dentistry, notably in restorative dentistry, implants, endodontics, and various methods of periodontitis therapy, including bone tissue engineering and medication administration. As we’ve shown in this review, employing this substance in conjunction with synthetic dental materials might boost its bacteriostatic or mycostatic properties. However, the main challenge is bringing in the aforementioned biomaterials. According to current studies, higher usage of chitosan in these regions will be favorable in terms of therapeutic success. Nonetheless, more research on the beneficial usage of chitosan is required.

## Figures and Tables

**Table 1:** Applications of Chitosan in diverse areas of dentistry.

Serial No.	Dental Arena	Chitosan Application	References
1	Preventive dentistry	Dentifrices, mucoadhesives, mouthwashes, antimicrobial agents, oral drug delivery	[26,27,28,36]
2	Conservative dentistry	Indirect pulp capping, direct pulp capping, pulpotomy, component of canal medicaments, sealants, enamel repair and remineralization, bonding agents	[5,7,29,30,41]
3	Surgery	Guided bone regeneration, hemostasis, bone tissue engineering, scaffolds	[35,36,42,43,44,45]
4	Implants	Titanium coatings along with chitosan, GC-based implants, bone regeneration around implants, peri-implantitis	[7,46,47,48,49,50,51]
5	Wound healing	Immune-modulators, gauze dressings	[2,4,36,43,44,45]

**Table 2:** Summary of available systematic reviews in the literature related to the use of chitosan in dentistry.

S. No	Authors Name	Type of Studies Included	No. of Studies Included	Important Findings
1	Cicciù, M., Fiorillo, L., & Cervino, G. 2019 [65]	Randomized controlled trials	12	Chitosan serves a variety of purposes, and it is employed in several dental specialties in a secure and efficient manner. Chitosan has a number of functions, including its ability to remineralize tooth tissue and, as a result, play the role of a desensitizer in toothpaste. Our comprehensive review found that using chitosan improved the surgical healing of oral ulcers sustained during tooth extraction. Additionally, when utilized in dental cement, some studies indicate a decrease in bacterial biofilm. Additionally, it has systemic qualities that make it useful for medication delivery, including antibacterial, antifungal, hemostatic, and other properties.
2	Liu, Ying M.S.; Chen, JiaLi M.S., R.N.; Li, PeiFang M.S.N., R.N.; Ning, Ning M.S., R.N. 2021 [1]	Randomized controlled trials	5	There have been more tests of novel chitosan dressings. However, there haven’t been many studies on how chitosan affects wound healing. According to recent research, chitosan does not impede the healing of wounds. The limited number of trials, however, made it difficult to interpret the data properly. To validate any clinically significant effect of chitosan on wound healing, further study must be carefully planned.
3	Pandiyan, I., Rathinavelu, P., Arumugham, M.I., et al. 2021 [68]	Randomized controlled trials	3	The most efficient chemical method of preventing plaque is mouthwash, which is used everywhere. Possible adverse effects include darkening of the teeth and tongue, a brief alteration in taste perception, a rise in calculus deposits, a burning sensation, and genotoxicity of buccal epithelial cells. In this review, the effectiveness of chitosan mouthwash in preventing plaque buildup and gingival irritation.
4	López-Valverde, N., López-Valverde, A., Ramírez, J.M. 2021 [69]	In vivo studies;Studies where at least one layer of CS was used to coat the Ti;Studies where bone growth or the formation of a biological seal around the Ti implant surface coated with CS alone or in combination with other products or molecules was assessed; Studies on endosseous implants; Studies that included non-modified animals (osteoporotics, diabetics…)	7	Ti dental implants with CS coating may be more capable of osseointegrating. The biofunctionalization of dental implants is probably going to become a commercial option in the future. However, to support the use of CS as a coating for Ti implants for osteoinduction purposes and subsequently to provide surfaces that ensure rapid osseointegration, confirmation of this possibility would require well-designed clinical research using broad samples, standardised protocols, and long-term monitoring.Ti dental implants with CS coating may be more capable of osseointegrating. The biofunctionalization of dental implants is probably going to become a commercial option in the future. To justify the use of CS as a coating, however, proof of this possibility would require well-designed clinical study involving large samples, standardised techniques, and long-term monitoring.
5	Lima, B.V., Oliveira, M.J., Barbosa, M.A., Gonçalves, R.M., & Castro, F. 2021 [70]	in vitro, in vivo and clinical studies which used Ch-based formulations and evaluated their ability to induce immune cell stimulation in the cancer context.	57	In general, Ch-based formulations reduce the number of cells that have anti-inflammatory effects while increasing the recruitment and proliferation of cells linked to pro-inflammatory properties. These outcomes were associated with a smaller tumour, fewer metastases, reversal of the immunosuppressive TME, and improved in vivo survival. Ch-based formulations, in general, present the possibilities for cancer immunotherapy. Clinical translation is still difficult, though, as most studies combine Ch with other ingredients, suggesting that part of the observed effects may be the consequence of the interaction of the separate effects.

## Data Availability

The authors confirm that the data supporting the findings of this study are available within the articles and can be shared upon request.

## References

[1] Liu X., Ma L., Mao Z., Gao C., Jayakumar R., Prabaharan M., Muzzarelli R.A.A. (2011). Chitosan-based biomaterials for tissue repair and regeneration. Chitosan for biomaterials II. Advances in Polymer Science.

[2] Tang G., Tan Z., Zeng W., Wang X., Shi C., Liu Y., He H., Chen R., Ye X. (2020). Recent advances of chitosan-based injectable hydrogels for bone and dental tissue regeneration. Front. Bioeng. Biotechnol..

[3] Younes I., Rinaudo M. (2015). Chitin and chitosan preparation from marine sources. Structure, properties and applications. Mar. Drugs.

[4] Zhu K.Y., Merzendorfer H., Zhang W., Zhang J., Muthukrishnan S. (2016). Biosynthesis, turnover, and functions of chitin in insects. Annu. Rev. Entomol..

[5] Merzendorfer H. (2011). The cellular basis of chitin synthesis in fungi and insects: Common principles and differences. Eur. J. Cell Biol..

[6] Finke M.D. (2007). Estimate of chitin in raw whole insects. Zoo Biol..

[7] Blumenthal H.J., Roseman S. (1957). Quantitative estimation of chitin in fungi. J. Bacteriol..

[8] Ifuku S., Nomura R., Morimoto M., Saimoto H. (2011). Preparation of chitin nanofibers from mushrooms. Materials.

[9] Nitschke J., Altenbach H., Malolepszy T., Mölleken H. (2011). A new method for the quantification of chitin and chitosan in edible mushrooms. Carbohydr. Res..

[10] Wu S., Zhou Y., Yu Y., Zhou X., Du W., Wan M., Fan Y., Zhou X., Xu X., Zheng L. (2019). Evaluation of chitosan hydrogel for sustained delivery of VEGF for odontogenic differentiation of dental pulp stem cells. Stem Cells Int..

[11] Ducret M., Montembault A., Josse J., Pasdeloup M., Celle A., Benchrih R., Mallein-Gerin F., Alliot-Licht B., David L., Farges J.C. (2019). Design and characterization of a chitosan-enriched fibrin hydrogel for human dental pulp regeneration. Dent. Mater..

[12] Singh G., Jamwal U. (2018). Chitosan hydrogel: Its applications in medicine and dentistry. Int. J. Prev. Clin. Dent. Res..

[13] Moreira M.S., Sarra G., Carvalho G.L., Gonçalves F., Caballero-Flores H.V., Pedroni A.C., Lascala C.A., Catalani L.H., Marques M.M. (2021). Physical and biological properties of a chitosan hydrogel scaffold associated to photobiomodulation therapy for dental pulp regeneration: An in vitro and in vivo study. BioMed Res. Int..

[14] Zhu Y., Zhang Y., Zhou Y. (2022). Application progress of modified chitosan and its composite biomaterials for bone tissue engineering. Int. J. Mol. Sci..

[15] Ma W., Zhang S., Xie C., Wan X., Li X., Chen K., Zhao G. (2022). Preparation of High Mechanical Strength Chitosan Nanofiber/NanoSiO2/PVA Composite Scaffolds for Bone Tissue Engineering Using Sol–Gel Method. Polymers.

[16] Thambiliyagodage C., Jayanetti M., Mendis A., Ekanayake G., Liyanaarachchi H., Vigneswaran S. (2023). Recent Advances in Chitosan-Based Applications—A Review. Materials.

[17] Riva R., Ragelle H., des Rieux A., Duhem N., Jérôme C., Préat V., Jayakumar R., Prabaharan M., Muzzarelli R.A.A. (2011). Chitosan and chitosan derivatives in drug delivery and tissue engineering. Chitosan for biomaterials II. Advances in Polymer Science.

[18] VandeVord P.J., Matthew H.W.T., DeSilva S.P., Mayton L., Wu B., Wooley P.H. (2002). Evaluation of the biocompatibility of a chitosan scaffold in mice. J. Biomed. Mater. Res..

[19] Konovalova M.V., Markov P.A., Durnev E.A., Kurek D.V., Popov S.V., Varlamov V.P. (2017). Preparation and biocompatibility evaluation of pectin and chitosan cryogels for biomedical application. J. Biomed. Mater. Res. Part A.

[20] Hoveizi E., Naddaf H., Ahmadianfar S., Gutmann J.L. (2023). Encapsulation of human endometrial stem cells in chitosan hydrogel containing titanium oxide nanoparticles for dental pulp repair and tissue regeneration in male Wistar rats. J. Biosci. Bioeng..

[21] Velasquez C.L. (2003). Algunos usos del quitosano en sistemas acuosos. Rev. Iberoam. Polímeros.

[22] Nadia M.C., Eric L., Giangiacomo T., Grégorio C. (2019). Fundamentals and Applications of Chitosan. Sustain. Agric. Rev..

[23] Parhi B., Bharatiya D., Purohit S.S., Swain S.K. (2023). Chitosan-Based Nano Biomaterials and Their Applications in Dentistry. Chitosan Nanocomposites: Bionanomechanical Applications.

[24] Noohi P., Abdekhodaie M.J., Saadatmand M., Nekoofar M.H., Dummer P.M. (2023). The development of a dental light curable PRFe-loaded hydrogel as a potential scaffold for pulp-dentine complex regeneration: An in vitro study. Int. Endod. J..

[25] Leite Y.K., Oliveira A.C., Quelemes P.V., Neto N.M., Carvalho C.E., Rodrigues H.W.S., Alves M.M., Carvalho F.A., Arcanjo D.D., Silva-Filho E.C. (2023). Novel Scaffold Based on Chitosan Hydrogels/Phthalated Cashew Gum for Supporting Human Dental Pulp Stem Cells. Pharmaceuticals.

[26] Zhang C., Hui D., Du C., Sun H., Peng W., Pu X., Li Z., Sun J., Zhou C. (2021). Preparation and application of chitosan biomaterials in dentistry. Int. J. Biol. Macromol..

[27] Celesti C., Iannazzo D., Espro C., Visco A., Legnani L., Veltri L., Visalli G., Di Pietro A., Bottino P., Chiacchio M.A. (2022). Chitosan/POSS Hybrid Hydrogels for Bone Tissue Engineering. Materials.

[28] Muzzarelli R.A.A., Muzzarelli C. (2005). Chitosan chemistry: Relevance to the biomedical sciences. Polysacch. I Struct. Charact. Use.

[29] Muzzarelli R.A.A., Muzzarelli C. (2009). Chapter 31: Chitin and chitosan hydrogels. Handbook of Hydrocolloids.

[30] Aranaz I., Mengíbar M., Harris R., Paños I., Miralles B., Acosta N., Galed G., Heras A. (2009). Functional Characterization of Chitin and Chitosan. Curr. Chem. Biol..

[31] Chatelet C., Damour O., Domard A. (2001). Influence of the degree of acetylation on some biological properties of chitosan films. Biomaterials.

[32] Ing L.Y., Zin N.M., Sarwar A., Katas H. (2012). Antifungal Activity of Chitosan Nanoparticles and Correlation with Their Physical Properties. Int. J. Biomater..

[33] Hafdani F.N., Sadeghinia N. (2011). A Review on Application of Chitosan as a Natural Antimicrobial, World Academy of Science. Eng. Technol..

[34] Mohire N.C., Yadav A.V. (2010). Chitosan-based polyherbal toothpaste: As novel oral hygiene product. Indian J. Dent. Res..

[35] De Carvalho M., Stamford T., Pereira E., Dos Santos P., Sampaio F. (2011). Chitosan as an oral antimicrobial agent. Formatex.

[36] Dilamian M., Montazer M., Masoumi J. (2013). Antimicrobial electrospun membranes of chitosan/poly(ethylene oxide) incorporating poly(hexamethylenebiguanide) hydrochloride. Carbohydr. Polym..

[37] Yadav A., Bhise S. (2004). Chitosan: A potential biomaterial effective against typhoid. Curr. Sci..

[38] Grobler S.R., Perchyonok V.T., Mulder R., Moodley D. (2015). Towards Bioactive Dental Restorative Materials with Chitosan and Nanodiamonds: Evaluation and Application. Int. J. Dent. Oral Sci..

[39] Croisier F., Jerome C. (2013). Chitosan-based biomaterials for tissue engineering. Eur. Polym. J..

[40] Chen X., Liu C., Liu C., Meng X., Lee C.M., Park H. (2006). Preparation and biocompatibility of chitosan microcarriers as biomaterial. Biochem. Eng. J..

[41] Kmiec M., Pighinelli L., Tedesco M.F. (2017). Chitosan-properties and applications in dentistry. Adv Tissue Eng. Regen. Med. Open Access.

[42] Ganss C., Von Hinckeldey J., Tolle A., Schulze K., Klimek J., Schlueter N. (2012). Efficacy of the stannous ion and a biopolymer in toothpastes on enamel erosion/abrasion. J. Dent..

[43] Nimbeni S.B., Nimbeni B.S., Divakar D.D. (2021). Role of Chitosan in Remineralization of Enamel and Dentin: A Systematic Review. Int. J. Clin. Pediatr. Dent..

[44] Schlueter N., Klimek J., Ganss C. (2013). Randomised in situ study on the efficacy of a chitin/chitosan toothpaste on erosive-abrasive enamel loss. Caries Res..

[45] Ebrahimi N., Soleimani A.A., Rashidiani J., Malekafzali B., Abedini F., Hosseinkhani H. (2019). Chitosan/fluoride nanoparticles for preventing dental caries. Curr. Dent..

[46] Resende A.H.M., Farias J.M., Silva D.D.B., Rufino R.D., Luna J.M., Stamford T.C.M., Sarubbo L.A. (2019). Application of biosurfactants and chitosan in toothpaste formulation. Colloids Surf. B Biointerfaces.

[47] Pavez L., Tobar N., Chacón C., Arancibia R., Martínez C., Tapia C., Pastor A., González M., Martínez J., Smith P.C. (2018). Chitosan-triclosan particles modulate inflammatory signaling in gingival fibroblasts. J. Periodontal Res..

[48] Hussain B., Karaca E.O., Kuru B.E., Gursoy H., Haugen H.J., Wohlfahrt J.C. (2021). Treatment of residual pockets using an oscillating chitosan device versus regular curettes alone—A randomized, feasibility parallel-arm clinical trial. J. Periodontol..

[49] Decker E.M., Weiger R., Weich L., Heide P.E., Brecx M. (2003). Comparision of antiadhesive and antibacterial effects of antiseptics on Streptococcus sanguinis. Eur. J. Oral Sci..

[50] Samprasit W., Kaomongkolgit R., Sukma M., Rojanarata T., Ngawhirunpat T., Opanasopit P. (2015). Mucoadhesive electrospun chitosan-based nanofiber mats for dental caries prevention. Carbohydr. Polym..

[51] Matsunaga T., Yanagiguchi K., Yamada S., Ohara N., Ikeda T., Hayashi Y. (2006). Chitosan monomer promotes tissue regeneration on dental pulp wounds. J. Biomed. Mater. Res. Part A.

[52] Hamilton M.F., Otte A.D., Gregory R.L., Pinal R., Ferreira-Zandona A., Bottino M.C. (2015). Physicomechanical and antibacterial properties of experimental resin-based dental sealants modified with nylon-6 and chitosan nanofibers. J. Biomed. Mater. Res. B Appl. Biomater..

[53] Park Y.J., Lee Y.M., Park S.N., Sheen S.Y., Chung C.P., Lee S.J. (2000). Platelet derived growth factor releasing chitosan sponge for periodontal bone regeneration. Biomaterials.

[54] ElShiha H.Y., Tawfik H.A.M., AbouSamrah N.K., Marzouk H.A.E.M. (2012). Efficacy of chitosan and absorbable gelatin sponge on hemostasis and wound healing following tooth extraction “A Comparative Study”. Egypt. Dent. J..

[55] Malmquist J.P., Clemens S.C., Oien H.J., Wilson S.L. (2008). Hemostatic of oral surgery wounds with the HemCon dental dressing. J. Oral Maxillofac. Surg..

[56] Tavaria F.K., Costa E.M., Pina-Vaz I., Carvalho M.F., Pintado M.M. (2013). A quitosanacomo biomaterial odontológico: Estado da arte. Rev. Bras. Eng. Biomédica.

[57] Ishihara M., Ono K., Sato M., Nakanishi K., Saito Y., Yura H., Matsui T., Hattori H., Fujita M., Kikuchi M. (2001). Acceleration of wound contraction and healing with a photocrosslinkable chitosan hydrogel. Wound Repair Regen..

[58] Ishihara M., Nakanishi K., Ono K., Sato M., Kikuchi M., Saito Y., Yura H., Matsui T., Hattori H., Uenoyama M. (2002). Photocrosslinkable chitosan as a dressing for wound occlusion and accelerator in healing process. Biomaterials.

[59] Mohabatpour F., Yazdanpanah Z., Papagerakis S., Chen X., Papagerakis P. (2022). Self-crosslinkable oxidized alginate-carboxymethyl chitosan hydrogels as an injectable cell carrier for in vitro dental enamel regeneration. J. Funct. Biomater..

[60] Sun Y., Miao T., Wang Y., Wang X., Lin J., Zhao N., Hu Y., Xu F.J. (2023). A natural polyphenol-functionalized chitosan/gelatin sponge for accelerating hemostasis and infected wound healing. Biomater. Sci..

[61] Kong M., Chen X.G., Xing K., Park H.J. (2010). Antimicrobial properties of chitosan and mode of action: A state of the art review. Int. J. Food Micro..

[62] Park S., Kim H., Choi K.S., Ji M.-K., Kim S., Gwon Y., Park C., Kim J., Lim H.-P. (2020). Graphene–Chitosan Hybrid Dental Implants with Enhanced Antibacterial and Cell-Proliferation Properties. Appl. Sci..

[63] Cicciù M., Fiorillo L., Cervino G. (2019). Chitosan Use in Dentistry: A Systematic Review of Recent Clinical Studies. Mar. Drugs.

[64] Sharifianjazi F., Khaksar S., Esmaeilkhanian A., Bazli L., Eskandarinezhad S., Salahshour P., Sadeghi F., Rostamnia S., Vahdat S.M. (2022). Advancements in fabrication and application of chitosan composites in implants and dentistry: A review. Biomolecules.

[65] Hallmann L., Gerngroß M.D. (2022). Chitosan and its application in dental implantology. J. Stomatol. Oral Maxillofac. Surg..

[66] Liu Y., Chen J., Li P., Ning N. (2021). The Effect of Chitosan in Wound Healing: A Systematic Review. Adv. Ski. Wound Care.

[67] Han B., Cao C., Wang A., Zhao Y., Jin M., Wang Y., Chen S., Yu M., Yang Z., Qu X. (2023). Injectable Double-Network Hydrogel-Based Three-Dimensional Cell Culture Systems for Regenerating Dental Pulp. ACS Appl. Mater. Interfaces.

[68] Pandiyan I., Rathinavelu P.K., Arumugham M.I., Srisakthi D., Balasubramaniam A. (2022). Efficacy of Chitosan and Chlorhexidine Mouthwash on Dental Plaque and Gingival Inflammation: A Systematic Review. Cureus.

[69] López-Valverde N., López-Valverde A., Ramírez J.M. (2021). Systematic review of effectiveness of chitosan as a biofunctionalizer of titanium implants. Biology.

[70] Lima B.V., Oliveira M.J., Barbosa M.A., Goncalves R.M., Castro F. (2022). Harnessing chitosan and poly-(γ-glutamic acid)-based biomaterials towards cancer immunotherapy. Mat. Today Adv..

